# The Infirmary Medical Superintendents' Society

**Published:** 1891-01-10

**Authors:** 


### THE INFIRMARY MEDICAL SUPERINTENDENTS' SOCIETY.

The monthly meeting of this society was held on Saturday, December 13th, at three p.m., at the Paddington Infirmary, when Dr. Savill, the medical superintendent of the establishment, showed the following interesting cases in the wards: The first case was that of a boatman, aged 29, with "mitral stenosis," illustrating the action of digitalis. The patient had been in the infirmary a year, and had gone through all the three stages of mitral obstruction, at first a thrill, then a presystolic murmur, now the murmur had almost disappeared. He was having ten minims of the tincture of digitalis every four hours, and the pulse, which had been rapid and irregular, was now quiet. Four cases of eczema were shown for suggestions as to treatment. In the discussion of these cases, Dr. Colcott Fox, who was present, said he was certain that nothing could prevent the tendency to relapse in old eczematous subjects. The treatment was founded on one or two simple rules, namely, that in all acute cases of eczema, soothing remedies should be applied, such as cold cream or oleate of bismuth. When the eczema becomes chronic, stimulating remedies were indicated, such as carbolic acid, resorcin, ithyol, tar, salicylic acid. Unna paints all his patients over night and morning with ithyol. This authority also says that all eczemas are parasitic in nature. There are many hundreds of dermatological floræ, which merely require a proper soil, produced possibly, for instance, by gout or dyspepsia; and the floræ readily grow and excite inflammation. He himself believed that post-eczematous-ithyosis occurred secondarily to any hypertrophy of the limbs. Mr. Lunn mentioned that he had known several cases of eczema break out together, two of which died of erysipelas; he found these cases were most relieved by hot baths. Dr. Gross recommended the use of Lassar's ointment in irritable cases. Dr. Larder had found dry boracic powder most useful. Another case shown was that of male hysteria with vaso-motor phenomena, hyper-tonicity and neuralgiæ. The patient was 61 years of age, who had been more or less paralysed for 24 years in the right leg and eight years in the left, and had been in the infirmary before. After treatment he had left the infirmary cured, but recently returned with the above-mentioned signs. Dr. Savill said the points of interest in male hysteria, as distinguished from the disease in the other sex, were as follows: 1, They were all of a melancholy type. 2, A history of heredity, generally from the mother. 3. Its inveterateness and tendency to relapse. As for treatment, he recommended isolation, cold shower baths, and the use of the faradic brush. Dr. Webster suggested the relation of this somna form of hysteria to hypochondriasis, and was due to something wrong with the digestion, frequently flatulence. Dr. Lloyd Tuckey, who was present, referred to a man with partial paraplegia who was prostrated from work, and had been at most of the hospitals without deriving much benefit. He hypnotised him, and suggested that he was able to walk. After the second sitting he got up and walked. He, however, returned after six months in the same condition, when the medical man, under whose care he was, suggested sending for Dr. Lloyd Tuckey again. The next day he was able to walk. Another case shown was that of tetanus, which had been cured by chloral, and which had been referred to at the last meeting of the society. In this case the median nerve of the injured arm had been divided by operation, and was followed by atrophy of the muscles and bones. Four months after, a neuroma was removed from the cicatrix. The patient had slowly recovered all the functions of the hand, but now complained of tenderness over the course of the nerve. Another case shown was that of purpura senilis occurring in a patient with rheumatic arthritis combined with albuminuria and œdema, and who had been treated with nitro-glycerine. The President considered the condition of the skin to be a local manifestion of the arterial changes of old age, which change in some cases gave rise to headache, giddiness, and finally necrosis of a part of the brain. Dr. Larder referred to patients getting these conditions when lying in bed through paralysis. It was a very important condition in respect to its appearance to bruises, for the production of which the attendants were sometimes apt to be blamed. A case of blenorrhagic arthritis and one of obesity, the latter treated on the meat and hot water system, were also shown. Dr. Lloyd Tuckey performed some important hypnotic experiments, a description of which we gave on page 202 of the issue of December 27th.

